# Fluorophores Use in Pituitary Surgery: A Pharmacokinetics and Pharmacodynamics Appraisal

**DOI:** 10.3390/brainsci11050565

**Published:** 2021-04-28

**Authors:** Daniele Bongetta, Fulvio Tartara, Fabio Pagella, Teresa Somma, Marilou Cavaliere, Giuseppe Di Perna, Francesco Zenga, Fabio Cofano, Diego Garbossa, Cesare Zoia

**Affiliations:** 1 Neurosurgery Unit, Ospedale Fatebenefratelli-Sacco, 20131 Milano, Italy; danielebongetta@hotmail.com (D.B.); mariloucavaliere@yahoo.it (M.C.); 2 Neurosurgery Unit, Istituto Clinico Città Studi, 20131 Milano, Italy; tartarafulvio@gmail.com; 3 Department of Otorhinolaryngology, Fondazione IRCCS Policlinico San Matteo, 27100 Pavia, Italy; f.pagella@smatteo.pv.it; 4 Department of Neurosciences and Reproductive and Odontostomatological Sciences, Division of Neurosurgery, Università degli Studi di Napoli Federico II, 80131 Napoli, Italy; teresa.somma85@gmail.com; 5 Neurosurgery Department, University degli Studi Milano Bicocca, 20126 Milano, Italy; 6 Neurosurgery Unit, AOC Città della Scienza e della Salute, 10127 Turin, Italy; zengafra@hotmail.com (F.Z.); dgarbossa@gmail.com (D.G.); 7 Department of Neuroscience “Rita Levi Montalcini”, University of Turin, 10127 Turin, Italy; fabio.cofano@gmail.com; 8 Neurosurgery Unit, Fondazione IRCCS Policlinico San Matteo, 27100 Pavia, Italy; gioiaoffice@gmail.com

**Keywords:** pituitary surgery, 5-ALA, ICG, fluorescein

## Abstract

(1) Background: Despite many surgical and technological advances, pituitary adenoma surgery is still burdened by non-negligible rates of incomplete tumor resection, mainly due to difficulties in differentiating pathology from normal pituitary tissue. Some fluorescent agents have been recently investigated as intraoperative contrast agents in pituitary surgery. The aim of this study is to evaluate the actual knowledge about the usefulness of such fluorophores with a particular focus on both the pharmacokinetics and pharmacodynamics issues of the pituitary gland. (2) Methods: We reviewed the current literature about fluorophores use in pituitary surgery and reported the first fully endoscopic experience with fluorescein. (3) Results: The studies investigating 5-ALA use reported contrasting results. ICG showed encouraging results, although with some specificity issues in identifying pathological tissue. Low-dose fluorescein showed promising results in differentiating pathology from normal pituitary tissue. Apart from the dose and timing of administration, both the fluorophores’ volume of distribution and the histological variability of the interstitial space and vascular density played a crucial role in optimizing intraoperative contrast enhancement. (4) Conclusions: Both pharmacokinetics and pharmacodynamics issues determine the potential usefulness of fluorophores in pituitary surgery. ICG and fluorescein showed the most promising results, although further studies are needed.

## 1. Introduction

Pituitary adenomas (PAs) are the third most common intracranial neoplasm, although roughly only 10% are symptomatic [1]. Apart from prolactin-secreting tumors, resection is the elective initial treatment, with good results in terms of clinical and endocrinological improvements [2]. Despite many surgical advances, such as the endoscope and neuronavigation, complete tumor removal is achieved only in about 80% of patients, whereas regrowth after an incomplete tumor removal may occur in up to 75% of cases [2,3]. Failure of surgery in macroadenomas is mainly due to the difficulty in visualizing the pathological tissue invading adjacent structures, such as the cavernous sinus or the suprasellar region. On the other hand, the intrapreoperative visualization for microadenoma lesions could be challenging, especially in those cases with difficult identification of the lesion even in magnetic resonance imaging (MRI). Therefore, new tools are still needed for a better localization of pathological tissues, so as to improve their selective removal and the preservation of pituitary functions. Intraoperative MRI has been proposed in order to decrease the rate of incomplete resections, but it is an expensive, time-consuming technique [4]. Since fluorescent agents such as 5-ALA, ICG, or fluorescein are gradually entering the neurosurgical practice as intraoperative contrast agents for cerebral neoplasm, some reports have been published about the use of fluorophores in pituitary surgery in recent years [5]. Of relevance, these fluorophores have different pharmacokinetic and pharmacodynamic features which have an impact on the contrast enhancing patterns, especially during real-time visualization.

The aim of this study is to evaluate the clinical usefulness of fluorophores in pituitary surgery with a particular focus on both their pharmacokinetics and pharmacodynamics issues. We also report our experience with the use of fluorescein as a dynamic contrast enhancer during a fully endoscopic ACTH-secreting adenoma surgery.

## 2. Methods

In January 2021, we searched PubMed/MEDLINE, EMBASE, and the Cochrane Library electronically to find articles dealing with the clinical use of fluorophores in pituitary surgery. More specifically, we used this search strategy in PubMed: (((fluorophores pituitary) OR (5-ALA pituitary)) OR (ICG pituitary)) OR (fluorescein pituitary). Two independent researchers conducted literature searches to assess the effective relevance of a given article: if questions arose, consensus was reached through discussion with the senior author.

The data about the perfusion of PAs and normal tissue was also reviewed and analyzed in relation to the different pharmacokinetics and pharmacodynamics features of 5-ALA, ICG, and fluorescein.

**Figure 1:**
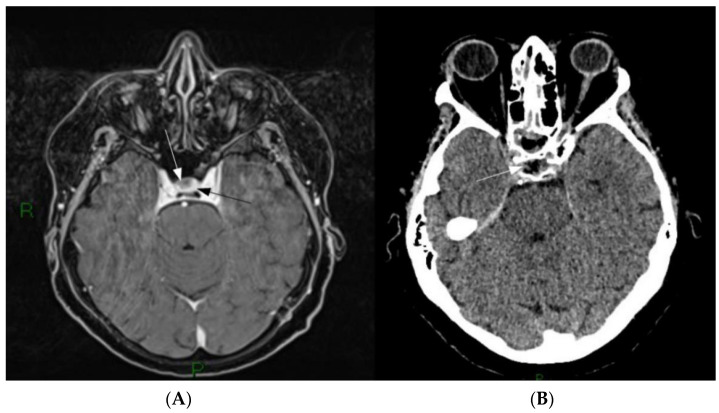
(**A**) Pre-operative Gd-MRI. A small, non-enhancing lesion is present on the right part of the sellar region (white arrow) surrounded by homogenously enhancing normal pituitary parenchyma (black arrow). (**B**) First day postoperative CT scan showing the ablation of the lesion (white arrow).

Moreover, the first case of ACTH-secreting pituitary adenoma treated by means of a fluorescein-aided fully endoscopic transnasal approach is described (Figure 1). 

**Figure 2:**
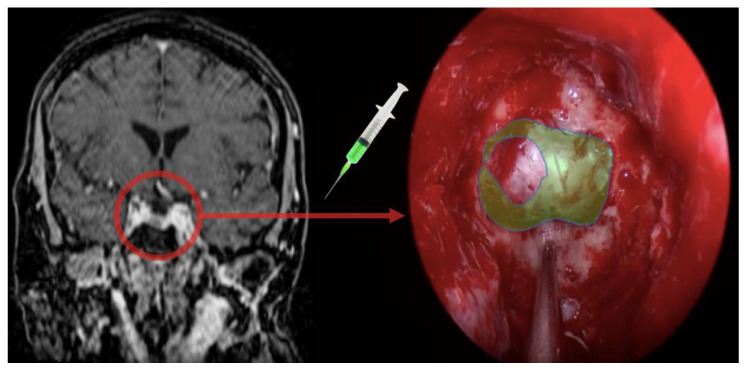
Rationale of the use of fluorescein as a gadolinium analog in real time visualization.

As for our fluorescein detection setup, we employed a customized filtering system as described elsewhere for both microscopic and endoscopic procedures [6,7]. Briefly, we employed a violet-blue light filter originally designed for endoscopic procedures, aiming at the detection of skull base cerebrospinal fluid (CSF) fistulas (Karl Storz GmbH & Co, 78532 Tuttlingen, Germany) on a 300-watt Xenon light source. We also employed a custom-designed yellow high-pass commercial photographic filter tailored to fit on top of the endoscope camera (XSOURCE), substituting for the proprietary one. The estimated filtering power was roughly half of that of the proprietary filter, thus enhancing real-time visualization while still allowing for fluorescein detection. After informed consent, the patient received a weight-based dose of 3 mg/kg bodyweight of sodium fluorescein (Monico SPA) administered via a peripheral vein line soon after the dural opening, following separate informed consent for this off-label application (Figure 2).

## 3. Results

**Figure 3:**
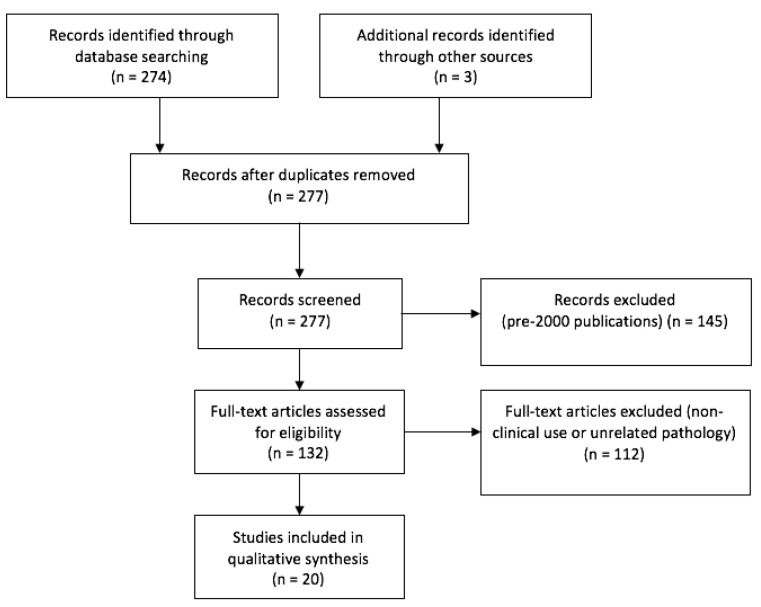
PRISMA flow chart.

The original query yielded 276 articles. The selection of meaningful papers, as per PRISMA guidelines, is outlined in Figure 3. The clinical experiences retrieved in literature, categorized by fluorophore agent, are reported below.

### 3.1. 5-ALA

5-ALA is a nonfluorescent endogenous compound that can be metabolized intracellularly into protoporphyrinogen IX (PpIX), a fluorescent intermediate product of the heme synthesis pathway. Its exogenous administration several hours prior surgery leads to selective accumulation of PpIX in some tumoral tissues such as malignant gliomas, although the exact mechanism responsible has yet to be determined. Few papers have investigated the clinical role of 5-ALA in pituitary surgery [8,9,10]. Eljamel and colleagues evaluated the use of intraoperative 5-ALA fluorescence to identify PAs with two different devices: an endoscopic photodiagnosis system and a laser-based probe for intraoperative spectrometry. The authors reported sensitivity and specificity of fluorescence endoscopy with 5-ALA of 80.8 and 75% respectively, whereas an approach with intraoperative spectrometry yielded 95.5 and 100%. These seemed to be promising results, albeit with some limitations. Firstly, no histological stratification of the results was reported, which could have shed light on the fluorescence pattern of the different pituitary adenomas. Another important issue was that they used a laser probe spectrometer instead of a standard blue light scope, thus limiting the clinical reproducibility of their findings. Lastly, it is not clear why 5-ALA should be more metabolized in adenomas: two further publications have investigated in vitro the use of 5-ALA fluorescence in PAs cells for photodynamic therapy, but this issue was not addressed [11,12].

Still, in a more recent paper, Marbacher et al., reported low rates or the absence of positive fluorescence among pituitary adenomas (8%, *n* = 1/12) using a standard blue light scope. In addition, a multicentric study on the use of 5-ALA during surgery for several skull base pathologies reported rather unfavorable results [10]. Noticeably, only 1 out of 15 PAs examined expressed a faint fluorescence, which led the Authors to state that 5-ALA fluorescence has limited utility in the majority of endonasal skull-base surgeries.

### 3.2. ICG

ICG is a water-soluble dye that binds to albumin and distributes rapidly after intravenous injection. Pharmacodynamically, it has no interaction with brain cells since it is cleared from the bloodstream by the liver and then excreted unchanged into bile. ICG has been used in pituitary surgery with different purposes. The first is the real-time observation of sellar vessels, whether arterious (ICAs, vasa nervorum) or venous (cavernous sinuses) [13,14,15,16]. The second application is to distinguish the normal pituitary parenchyma from PAs via their different patterns of vascularization as revealed by ICG. In this particular regard, different pharmacokinetic approaches have been employed. Firstly, real-time administration has been tested—i.e., a bolus of ICG administered at the time of surgery. In the first two reports, twelve patients were evaluated with an endoscopic approach [17], whereas 22 were evaluated with a microsurgical one [18]. Generally, these authors deemed their techniques to be useful for the identification of the different structures. In particular, the most useful phase for the differentiation of the different structures was reportedly the flash-filling phase prior to the recirculation of the dye. Despite employing the same dosage (25 mg), Litvack et al. always reported a hypofluorescence of PAs as compared to normal pituitary gland tissue, irrespective of their histology, whereas Sandow et al., reported both 100% of hyperfluorescence of ACTH-secreting adenomas (6 out of 6) and a hypofluorescence in GH-secreting adenomas for 9 cases out of 13. Litvack et al., also reported a nodular hypervascularity of the sellar dura in all cases of prolactinoma and acromegaly (*n* = 4). These discrepancies are difficult to interpret: some differences may be ascribed to the different visualization systems. Sandow et al. acknowledged that their microscopic results showed a lower signal intensity due to the distance of the light source to the surgical field as compared with the endoscopic series [18]. Subsequently, several authors reported encouraging results with lower doses of ICG. For example, Verstegen et al. reported that the use of a fixed 10 mg dose of ICG may shorten the time of fluorescence fading as well as help to better understand the pharmacodynamics of contrast enhancement during different stages of the intervention [19]. Moreover, Amano et al. used further reduced fixed doses of 6.75 mg of ICG in order to show that PAs are more fluorescent than the normal anterior pituitary for the first 7 min, gradually becoming the opposite from the 8th min onward [20]. The repeated administration also showed that the normal gland improved its vascular perfusion after tumor removal, due to its decompression. Interestingly, after administering 12.5 mg of ICG twice an operation with an interval >30 min, Inoue et al. were also able to detect differences in fluorescence patterns related to tumor size [21]. Specifically, as tumor size increased, elapsed times to fluorescence detection for normal pituitary gland were prolonged, whereas those for the tumor were reduced. More recently, Shahein et al. tried to define indications and limitations for the use of ICG based on previous experience and their own experiences with 10 PA cases. In particular, they were the first to systematically correlate MRI images with intraoperative ICG fluorescence, postulating that the various patterns of enhancement seen in MRI are due to the differences between the vascular features of the gland and the tumor [22]. Starting from these assumptions, they indicated that both the presentation to the sellar surface, the presence of an intact pseudocapsule, and intratumoral bleeding were the main factors that should be considered when selecting cases.

Apart from this “on-the-spot” approach, a group from the University of Pennsylvania investigated another pharmacokinetic approach in several papers [23,24,25]. For example, they introduced the concept of second window ICG (SWIG) in pituitary surgery. First reported by Madajewski et al., this technique employs a high-dose infusion of ICG (5–7.5 mg/kg) given 24 h prior to surgery, in order to allow the dye to accumulate in areas of neoplasm [26]. The pharmacodynamics mechanism underlying the dye accumulation is likely the enhanced permeability and retention (EPR) effect, which implies that solid tumors possess enhanced vascular permeability due to defective endothelial structures, impaired lymphatic drainage systems, and increased permeability mediators [27]. The accumulation of ICG with this method is so diffuse that in Pas, SWIG demonstrated 100% sensitivity as well as up to 29% specificity. Therefore, one of the limitations of SWIG is the high false-positive tumor detection, which could come from other tissues associated with endothelial damage, such as inflammation and necrosis. In order to try to overcome this issue, the same group also introduced a pharmacodynamics variable, namely a new contrast agent capable of interacting specifically with tumor receptors. More specifically, they investigated OTL38 (On Target Laboratories, West Lafayette, Indiana), which is a folate analog conjugated to a cyanine dye that specifically binds folate receptors [28]. In fact, previous studies demonstrated that non-functioning adenomas (NFA) usually overexpress folate receptor alpha (FRα) compared to both a normal pituitary gland, hormone secreting functional Pas, and surrounding skull base structures [29]. OTL38 was administered at 0.025 mg/kg of bodyweight 3 h prior surgery and its usefulness has been studied in 14 NFA. Unfortunately, only 9 out of 14 patients demonstrated FRα-overexpression on immunohistochemistry. Nevertheless, NIR fluorescence showed a striking 100% sensitivity and specificity for pathological tissue in those actually FRα-overexpressing NFA. As the authors correctly reported, the FRα expression level of the adenomas, the endoscope-to-sellar distance, and the NIR hardware employed may have a significant impact on the fluorescent signal-to-background ratio. Besides the relative cost and rarity of the hardware employed, as well as the experimental setting in the use of OTL38, these issues limit the widespread use of this technique.

### 3.3. Fluorescein

Fluorescein is an oral or, most frequently, endovenous tracer. Once in the blood stream, it is metabolized to fluorescein monoglucuronide, excreted by the kidney and conjugated in the liver. It can also distribute well in the interstitial space, which is why it is used in high-grade glioma surgery: its accumulation in the interstitial areas of tumor-induced ruptured blood-brain barrier (BBB) depicts the borders of the lesion, similar to a gadolinium-enhanced MRI [30]. Nevertheless, specificity is not guaranteed, as fluorescein exists in both pure and protein-bound forms in the blood, the former being small enough to cross the intact BBB. To try to overcome these issues, the use of a pegylated versions of fluorescein showed a 38% improvement in tumor-to-normal contrast in preliminary studies [31].

In reviewing the topical literature, the first report on the use of fluorescein in a pituitary adenoma was a high-dose bolus (20 mg/kg) administered with the lesion in vision under simple microscopic white light [32]. This was reported to increase the identification of the adenoma. Unfortunately, no histological specification or relation to the normal pituitary tissue was reported.

The second experience with fluorescein was only recently published by Romano-Feinholz et al., employing a technique involving a hybrid endoscopic-microscopic surgical resection [33]. In particular, they performed a standard endoscopic approach and checked differences in pituitary tissues with the aid of a fluorescein-detecting microscope at the time of lesion visualization. All patients reporting fluorescence differences between PA (2 GH and 1 ACTH-secreting) and a normal pituitary gland also presented a relevant hyperintensity in the PA areas. They also showed that a visual difference exists between the fluorescence intensity of the PAs and that emitted by scar tissue in recurring tumor cases, which they deemed very useful in avoiding complications. Most noteworthy is that after their first experiences, they employed slightly higher doses than usual for gliomas (5 mg/kg) by administering a bolus of 8 mg/kg approximately 10 min before the durotomy [34].

**Figure 4:**
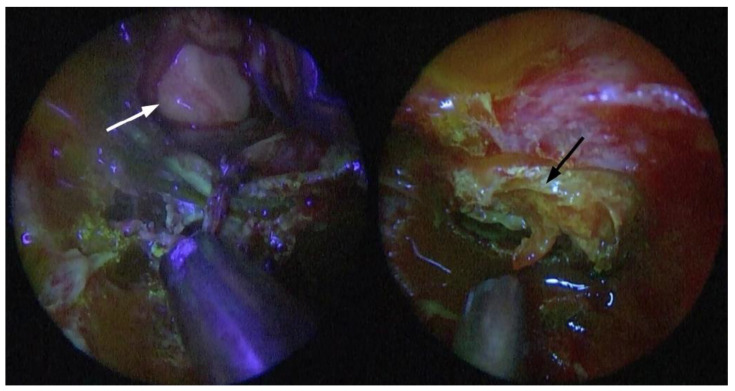
Endoscopic fluorescein-filtered vision of the sellar region; intradural stage, after adenoma isolation from normal parenchyma: non-enhancing adenoma (white arrow) and vividly enhancing pituitary parenchyma (black arrow).

On the contrary, our report represents the first fully endoscopic approach of fluorescein-aided pituitary surgery. Even our experience showed the ability for fluorescein to distinguish pathology from normal tissue, albeit in a completely inverted fashion—i.e., showing a relevant hypointensity in the PA after roughly 40 s (Figure 4). This may be due to the adoption of a different pharmacodynamics approach: first, we administered the bolus in vision of the pathology, not 10 min prior. This was planned in an attempt to mimic the preoperative diagnostic assessment with dynamic contrast enanched MRI sequences as much as possible. We know for a fact that there are either early or late contrast enhancing features that aid in the identification of the pathologic tissue depending on its histology. Since the entire gland shows a homogenous enhancement within 30–60 s, the maximum image contrast between the normal pituitary and microadenomas is soon after this timeframe, hence the usefulness of early MRI dynamic sequences after bolus administration [35]. Microadenomas, such as our typical ACTH case, usually appear as relatively nonenhancing lesions with regard to an intensely enhancing pituitary gland in these sequences. Several other MRI sequences, however, have revealed significant differences between pituitary tumors and normal gland tissue with respect to time sequences and enhancement patterns. In particular, there can be late (>10 min) reversal of the contrast enhancement in some pituitary adenomas with poor vascularization [36]. A second difference in our approach is that we chose to try to maximize the differences between enhancing and nonenhancing structures by lowering the dose administered, thus following the idea of using fluorescein as an intraoperative, visible gadolinium analog. More specifically, since the proportion of gland-to-lesion contrast enhancement remains stable with different dosages of gadolinium at about 26% [37], we administered a bolus of 3 mg/kg, which is approximately half of the dose used for glioma surgery in order to obtain a fluorescence detection more similar to an all-or-nothing setting. We also postulate that the low-dose detection has been possible because we were able to significantly reduce the distance from the exciting light source to the tissue examined with a fully endoscopic procedure. Starting from these assumptions, we have specifically designed an ongoing study in order to further correlate early and late gadolinium-enhanced MRI sequences of different histological cases with intraoperative fluorescein detection.

Fluorescein, however, proved itself useful not only in direct endoscopic vision of the pathological tissue, as in our experience, but also in intraoperative pathological examinations. In fact, the first experience of fluorescein-based confocal laser endomicroscopy (CLE) was recently published [38]. Albeit tested on only nine ex vivo samples, fluorescein provided sufficient contrast for CLE and proved to be a potential alternative to frozen section analyses. Interestingly, Belyk et al. also reported that the optimal timing for CLE imaging was determined to range between 1 min and 10 min after fluorescein injection, thus further corroborating the similarities with gadolinium pharmacokinetics.

## 4. Discussion

Pharmacodynamics is defined as the study of how drugs have effects on the body, the most common mechanism of interaction being with tissue receptors located either in cell membranes or in the intracellular fluid. Of the three fluorophores reviewed, only 5-ALA has a significant metabolic action, per se, at a cellular level, ICG and fluorescein being rather inert compounds. Still, the exact mechanism by which tumor tissue might metabolize 5-ALA at a higher rate is still unknown, even for other well-studied histologies, such as high-grade glioma [39]. Moreover, the fluorescence intensity in gliomas is known to be directly related to the rate of tumoral cellular turnover, which implies the absence of clinical use in low-grade tumors. Since human pituitary tumors typically show relatively modest increases in mitotic activity, we infer that this may similarly limit the actual usefulness of this fluorophore in pituitary surgery [40]. On the contrary, the specific pharmacodynamics of OTL38, a folate analog conjugated to a cyanine dye, are responsible for the excellent levels of specificity obtained in a subset on nonfunctioning PAs. In the future, selective over- or under-expression of membrane receptors specific for different histological tissues might hopefully be revealed, thus establishing the use of cyanine-conjugated drugs as one the most promising approaches for future research.

Pharmacokinetics, on the other hand, refers to the time course of drug absorption, bioavailability, distribution, metabolism, and excretion. The dose and rate of administration as well as the state of hydration, age, and renal function of the patient may hinder a clear-cut assessment. For instance, it has been demonstrated that the rate variation from 5 mL/s to 1 mL/s of contrast medium injections could actually imply around a triple time to peak (45 to 135 s) in the aortal compartment [41]. Moreover, several peculiarities of the pituitary tissue are to be taken into account. First, the pituitary gland resides outside of the blood-brain barrier, and contrast media can rapidly balance between the vascular and interstitial spaces. Second, there are significant differences in microvascular density between the normal anterior pituitary region and different PAs [42,43]. Third, the relative amount of interstitial space varies between different histological tissues [44]. Fourth, the vascular supply of adenomas is mainly arterial via neovascularization instead of a slow, portal vasculature of the normal anterior pituitary region [45,46,47,48]. Finally, all these variables are to be balanced with drug-specific pharmacodynamical features, in particular the volume of distribution (VD). VD represents the apparent volume into which the drug is distributed to provide the same concentration as it currently is in the blood plasma, thus reflecting the extent to which the drug is present in extravascular tissues. More specifically, ICG VD is 0.07 L/kg, whereas fluorescein VD is around 0.5 L/kg [49]. In comparison, the VD of gadolinium contrast-enhancing agents is around 0.25 L/kg, an interstitial space-permeation value rather similar to fluorescein. This implies that ICG tends to stay more inside the vessels, whereas both fluorescein and gadolinium tend to rapidly diffuse into the interstitial space, especially in slow-flow vascular districts such as the pituitary portal. Starting from these assumptions, we might be able to better interpret some of the data from literature. More specifically, we can understand why the contrast enhancing seems to fade or even invert as time passes, especially for ICG: as fluorophores diffuse from the vessels to the interstitial tissue, the microvascular component appears to be less evident; conversely, at later stages the contrast wash-out is enhanced in highly vascularized tissues, rendering the PA parenchyma more fluorescent. Moreover, the improved visualization reported with lower doses for both fluorophores may be justified by a more progressive reaching of homeostasis between intravascular and interstitial concentrations, thus fostering a more sensible visualization of the differences between enhancement patterns of tissues.

Despite these potentially useful considerations, all the studies reviewed, including our own experience, suffer from several limitations. One reason is that the number of patients studied is still low. Furthermore, different dosages or multiple subsequent administrations are employed even within the same study. Finally, a complete preoperative assessment is often lacking. 

## 5. Conclusions

Pharmacokinetics and pharmacodynamics issues determine the potential usefulness of fluorophores in pituitary surgery. Future research needs to systematically focus on these variables as well as on a rigorous analysis of both preoperative radiological imaging features, intraoperative findings (tumor volume, capsule integrity, microhemorrhages), and histological characteristics.

## Data Availability

Not applicable.
